# Reaction Mechanism of Etherification of Rice Straw with Epichlorohydrin in Alkaline Medium

**DOI:** 10.1038/s41598-019-50860-3

**Published:** 2019-10-04

**Authors:** Guanglu Li, Yan Shang, Yuhong Wang, Linshan Wang, Yuesheng Chao, Yang Qi

**Affiliations:** 1 College of Science, Northeastern University, Shenyang, 110819 China; 2 Institute of Heritage Conservation Theory and Technology, Jilin Jianzhu University, Changchun, 130118 China; 3 School of Materials Science and Engineering, Northeastern University, Shenyang, 110819 China

**Keywords:** Chemistry, Materials science

## Abstract

Wood plastic composites (WPCs) made from plant fibres and plastics have gained more and more attention. Studies have been focused on preparation and mechanical performance of WPCs. While mechanism of chemical modification of cereal straw has rarely been reported. In the present work, rice straw was etherified with epichlorohydrin (EPI) and the mechanism of etherification was investigated. Natural rice straw (NRS) was pretreated with NaOH to move most of hemicellulose and lignin. The alkali treated rice straw (ARS), whose dominant component being cellulose, was etherified with EPI at 120 °C for 1–8 h in toluene with NaOH as catalyst. NRS, ARS and etherified rice straw (ERS) were characterized and analyzed by FT-IR, solid CP/MAS ^13^C-NMR, elemental analysis and neutral sugar analysis. The etherification reaction was finished within 5 h, and C_3_H_6_O units were introduced into the structure of cellulose, leading to the increase of contents of C and H in ERS. The etherification process of ARS in alkaline medium was divided into three stages, during which two hydroxyl groups were replaced by two ether bonds successively, and a new hydroxyl group was formed in the last step. The number of hydroxyl groups in ERS was reduced, and reduction of hydrophilicity of ERS could be expected.

## Introduction

Plastics and their derivatives are widely used in the world. As a result, the difficult degradation of plastics has caused a series of environmental problems. Recently, with the continuous reduction of forest area and the depletion of oil-based resources, degradable wood plastic composites (WPCs) have gained more and more attention^1–4^. WPCs are mainly made from plant straws (or wood residues) and plastics by extrusion, injection or compression^2,5,6^.

The demand for WPCs in the world is increasing, with the global output exceeding 1.5 million tons^7^. WPCs can make full use of waste plastics and waste wood, by overcoming the shortcomings of poor mechanical strength and great deformability of woods, and low elastic modulus of plastics^7^. The application of WPCs can reduce environmental pollution of plastics.

Development of WPCs is of great significance for comprehensive utilization of agricultural and forestry wastes and waste plastics^8^. When plant fibers are embedded into the polymer matrix, absorption of moisture by the fiber is restricted, growth of fungi in woods is inhibited, and hence service life of WPCs is prolonged. Compared with inorganic fillers, plant fibers show advantages of biodegradability, low density, and low cost^9^. Thermoplastics used for preparation of WPCs are mainly polyethylene (PE), polypropylene (PP) and polyvinyl chloride (PVC). The main factors affecting the properties of WPCs are dispersion of plant fibers in the matrix and interfacial compatibility between plastics and plant fibers. It has been recognized that interfacial compatibility is the key problem^10^.

Plant fibers are mainly composed of cellulose, hemicellulose and lignin. There are a large number of hydroxyl groups which are of strong polarity in structure of cellulose and hemicellulose. So, cellulose and hemicellulose have strong polarity and hydrophilicity, and their compatibility with hydrophobic plastics is poor. In the process of blending and molding of WPCs, plant fibers lost their bound water due to temperature rise, resulting in microporosity and internal stress defects in the composite material^11^. Therefore, treatment of plant fibers is needed to reduce the polarity and hydrophilicity of plant fiber surface, enhance interfacial compatibility and thus improve the comprehensive properties of WPCs^12,13^.

General ways to improve the interfacial compatibility in WPC are addition of compatibilizer^14–17^, surface grafting of plastics^18–20^, and chemical modification of plant fibers^21,22^. The use of interfacial compatibilizer in WPCs can improve the compatibility of material interface to a certain extent, but cannot fundamentally overcome structural defects^11,13^. After chemical modification, such as etherification^23^ and esterification^1^, plant fibers exhibit hydrophobicity and thermoplasticity to certain extend, and their interface compatibility with resin matrix is improved^1,3,24,25^.

So far, researches related to WPCs have focused on modification of plant fibres^1,3,12,13^, surface grafting of matrix resins^18–20^, blending techniques^2,5,6^, and mechanical performance^1,2,5,6^. Studies on mechanism of the chemical modification of cereal straw have rarely been reported. In the present work, reaction mechanism of etherification of rice straw by epichlorohydrin (EPI) was investigated.

## Experimental Sections

### Materials

Rice straws were collected from local farm, which were cut into 2 cm sticks, and washed with tap water and followed by dring at 80 °C for later use. All chemicals were bought from Sinopharm Group Chemical Reagent (Shenyang) Co. Ltd., China, without further purification. Distilled water was used for preparing aqueous solutions or washing products.

### Pretreatment of rice straws

The dried rice straw sticks were grounded with a herbal medicine mill and then sieved through an 80 mesh (180 μm) sieve. The obtained natural rice straw powder was labeled as NRS. The NRS powder was boiled with 10% NaOH solution for 3 h, and washed with distilled water until neutral. The alkali treated NRS powders were dried at 60 °C in a vacuum oven, and labeled as ARS.

### Etherification of rice straws

6 mL of 50% NaOH solution and 20 mL of toluene were transferred to a 50 mL three-necked flask, which was loaded with 2 g of ARS powders. The mixture reacted at 120 °C for 30 min under stirring. Then 6 mL of EPI was added and continuously refluxed at 120 °C for 1~8 h. The product was recovered by suction filtration and then was washed with ethanol and distilled water until neutral. The washed 3 powders were dried at 60 °C in a vacuum oven. Then etherified ARS was obtained and labeled as ERS.

### Analysis of rice straws

Fourier transform infrared (FT-IR) spectra were obtained on an IR spectrometer (JASCO IR-615, Japan) with a KBr disc containing 1% finely ground samples. Scanning was conducted for 64 times and the wave number range from 400 cm^−1^ to 4000 cm^−1^ at a resolution of 4 cm^−1^ in the transmission mode (%). CP/MAS ^13^C NMR analysis of NRS and ERS was carried out on a nuclear magnetic resonance spectrometer (Infinity 300, Vavian Inc, USA), operationg at acceptance time of 12.800 ms, delay time of 4 s, frequency of 75.4 MHz, 90° pulse time of 2.30 ms, and scanning time of 60 min. Compositions of NRS, ARS and ERS (8 h) were measured by an elemental analyzer (2400 II, Perkin-Elmer Inc, USA). Process for analysis of neutral sugars is as follow^26^.

50 mg RS samples were added in 1 mL of 72% sulfuric acid and then a suspension was obtained by shaking in an oscillator (150 rpm) for 4 h at room temperature. The mixture was diluted to 18 mL and heated at 120 °C in a autoclave for 1 h for hydrolysis. The resulting solution was filtrated and transferred into an 100 mL volumetric flask and diluted to volume with distilled water. 5 mL of rice straw hydrolysate solution was mixed with 1 mL of standard inosite solution (1.00 mg/mL), and the mixture was neutralized to pH 5.5 by Ba(OH)_2_ solution (0.1 M). After centrifugation (4000 rpm), the supernatant was reduced by 20 mg of NaBH_4_ at room temperature overnight. Residual NaBH_4_ was removed by adding a drop of acetic acid. Vacuum distillation with methanol was carried out for 3 times. The distillate was heated at 105 °C in oven for 15 min, then 1 mL of acetic anhydride was added and reacted at 120 °C for 3 h. Contents of the reaction products were analyzed with a gas chromatograph (GC-14B, Shimaddzu Inc, Japan), equipped with a flame ionization detector, fused silica capillary column (Ф0.25 mm × 30 m, TC-17, GL Science, Japan), and H_2_ as carrier gas, running at heating rate of 0.5 °C/min, column temperature of 220 °C, injection temperature of 220 °C, and detector temperature of 230 °C.

## Results and Discussion

### FT-IR spectra

**Figure 1:**
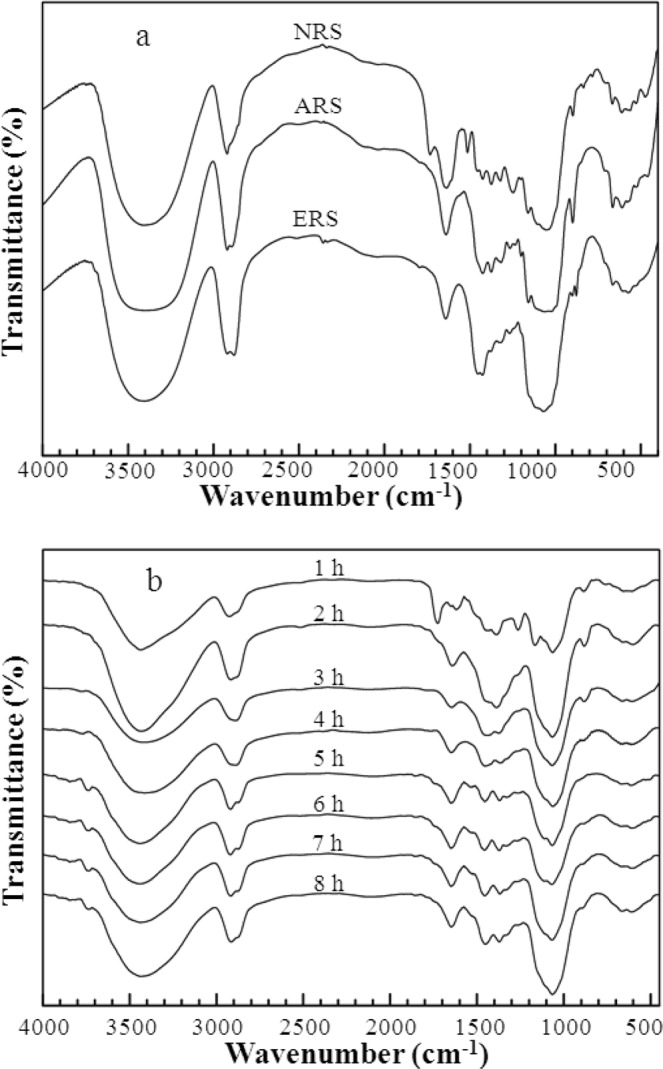
FT-IR spectra of (**a**) NRS, ARS and ERS (8 h) and (**b**) ERS with different reaction time.

Figure 1(a) shows the FT-IR spectra of NRS, ARS and ERS (8 h), and Fig. 1(b) shows the FT-IR spectra of ERS with different modification time (1 h~8 h). Peaks around 3400 cm^−1^ were of stretching vibration of hydroxyl in both rice straw and its adsorbed water. Peaks for stretching vibration and bending vibration of –CH_2_ located at 2900 cm^−1^ and 1427 cm^−1^, respectively. Peak at 1060 cm^−1^ were of stretching vibration of ether bond C–O–C. Peaks at 1060 cm^−1^ (C–O–C) and 2900 cm^−1^ (–CH_2_) in ERS were strengthened, demonstrating that quantity of groups of C–O–C and –CH_2_ increased after etherification by EPI. In the FT-IR spectrum of NRS, peak at 1734 cm^−1^ was the characteristic of hemicellulose^27^, peak at 1516 cm^−1^ was the characteristic of lignin^28^, both of which did not appear in the spectra of ARS and ERS, indicating that hemicellulose and lignin were removed by alkali treatment. Peak at 1638 cm^−1^, which was of bending vibration of hydrogen bonds of adsorbed water, of ERS was weaker than those of NAS and ARS. This phenomenon suggested that hydrophobicity of ERS enhanced and capacity to adsorb water reduced. Peaks around 890 cm^−1^ were of aliphatic carbocycle vibration of saccharides, such as mannose, rhamnose and arabinose in cellulose. Again peak strength of 890 cm^−1^ of ERS was weaker than that of ARS, promoting that the contents of saccharides in cellulose decreased by etherification. From Fig. 1(b) it can also be seen that saccharides peak at 890 cm^−1^ almost disappeared at 4 h and later, and peaks of ether bond (C–O–C) were strengthened after 5 h. The characteristic peaks of epoxy groups in the range of 1280~1180 cm^−1^ were notable at 1 h, but disappeared from 2 h. In all spectra of ERSs (1 h–8 h), characteristic peak of C-Cl bond in the range of 700~750 cm^−1^ did not appear. These facts implied that, in etherification with EPI, the C-Cl bond broke within 1 h, and the epoxy ring opened within 2 h. Wavenumbers and strength of peaks in the IR spectra of ERS from 5 h to 8 h were almost identical, demonstrating that the etherification process was finished within 5 h.

### Solid CP/MAS ^13^C-NMR

**Figure 2:**
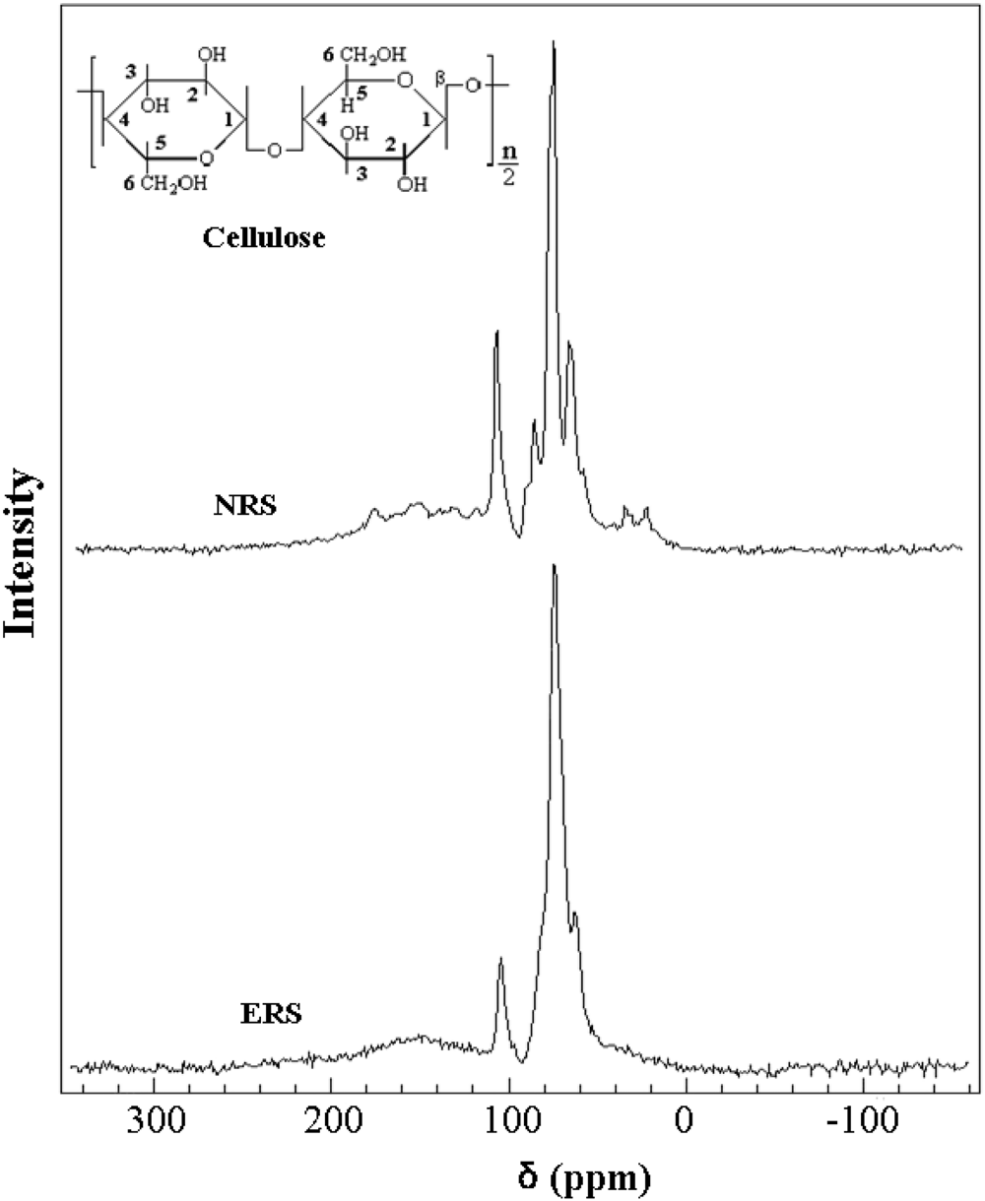
CP/MAS ^13^C-NMR spectrua of NRS and ERS (8 h).

Figure 2 shows the CP/MAS ^13^C-NMR spectra of NRS and ERS (8 h), inset being the structural formula of cellulose. Because NRS has a variety of ingredients and complex structure, its spectrogram consisted of several quite wide peaks which were difficult to be distinguished. Chemical shifts (δ) that located from δ 65.0 ppm ~ δ 110.0 ppm represented cellulose^11,29,30^. The signal of δ 105.6 ppm was referred as cellulose C-1. The signal of δ 87.9 ppm was referred to cellulose crystalline region C-4, while δ 84.4 ppm was referred to cellulose amorphous region C-4. Signals at δ 75.4 ppm and δ 73.3 ppm represented cellulose C-2, C-3 and C-5. Signal at δ 65.1 ppm represented cellulose C-6. Some of the weaker peaks were the characteristic absorption signals of hemicellulose and lignin. The characteristic chemical shifts of hemicellulose were of acetyl group at δ 22.0 ppm, and of carbonyl group at δ 173.0 ppm^29,30^. The signial at δ 56.8 ppm was methoxy group (-OCH_3_) in lignin structure, and signal of benzene ring in lignin structure is located at δ 153.2 ppm^29,30^.

After etherification by EPI, the absorption signal of C-4 in the cellulose crystalline region disappeared, indicating that etherification had led to decrystallization of cellulose. Because etherification could not take place at C-1, the chemical shift intensity of C-1 at 105.6 ppm would not change. Compared with C-1, the relative intensity of chemical shift signals for C-2, C-3, C-5 and C-6 became weaken, and the signal of C-6 weakened more. It could be supposed that the etherification reaction occurred in C-2, C-3, C-5 and C-6 positions. In the spectrum of ERS, characteristic absorption signals representing hemicellulose (at δ 22.0 ppm and δ 173.0 ppm) and lignin (at δ 56.8 ppm and 153.2 ppm)^29,30^ could not be observed, indicating that hemicellulose and lignin had been removed, matching with the result of FT-IR.

### Elemental analysis

Contents of elements C, H and N of NRS, ARS and ERS were analyzed by an elemental analyzer. Supposing inorganic impurities had been removed completely by alkali treatment^28,31^, ARS and ERS were composed of C, H, O and N. So, the contents of O in ARS and ERS were determined by the difference between total amount and the sum of C, H and N. Content of N might be referred to tiny amount of proteins in rice straw^31^. The results are listed in Table 1. Carbon content of ARS was less than that of NRS, because both hemicellulose and lignin with high carbon content were removed by alkali treatment^28,31^. Pure cellulose, whose molecular formula is (C_6_H_10_O_5_)_n_, consists of 44.44% (w/w) C and 6.17%(w/w) H. The chemical compositions of ARS were of 40.75%(w/w) C and of 6.05 &(w/w) H, both less than those of cellulose. After etherification by EPI, C_3_H_6_O unit was introduced into cellulose. In C_3_H_6_O unit, the contents of C and H were 62.07%(w/w) and 10.34%(w/w), respectively, both higher than those in ARS and pure cellulose. Supposing each hexatomic ring (C_6_H_10_O_5_) of cellulose was introduced with a unit of C_3_H_6_O by etherification with EPI, formula of modified cellulose would become C_9_H_16_O_6_, in which contents of C and H were 49.09%(w/w) and 7.27%(w/w), respectively. In ERS sample, contents of C and H were 45.01%(w/w) and 6.92%(w/w), respectively, both higher than those in ARS and less than those in theoretical C_9_H_16_O_6_. Compared with ARS, ERS had higher contents for both C and H, and less content for O.

**Table 1:** Composition of NRS, ARS and ERS.

Samples	Content(%(w/w))				C:H:O
	C	H	N	O^c^	
NRS	41.93	5.95	0.97	-^d^	—
ARS	40.75	6.05	0.18	53.02	1.02: 1.83: 1
ERS(8 h)	45.01	6.92	0.09	47.98	1.25: 2.31: 1
C_6_H_10_O_5_^a^	44.44	6.17	—	49.39	1.20: 2.00: 1
C_9_H_16_O_6_^b^	49.09	7.27	—	43.64	1.50: 2.67: 1

a-Unit of cellulose, theoretical data; b- Unit of etherified rice straw, theoretical data; c- Determined by subtracting the sum of C, H and N from 100 percent; d-The composition of NRS was complicated and content of O could not be estimated.

### Analysis of neutral sugars

The primary structure of hemicellulose is polyxylose, i. e., poly(arabino-4-O-methylglucuronidexylose). Cellulose is a polysaccharide composed of D-glucose rings linked by β 1—4 glycoside bonds. The main hydrolysates of hemicellulose and cellulose are xylose and glucose, respectively. Glucose and xylose could be used as index for contents of cellulose and hemicellulose in samples, respectively^11^. Table 2 lists the neutral sugar contents of hydrolysis products from NRS, ARS, and ERS (modified time from 1 h to 8 h). Six kinds of monosaccharides, i. e., rhamnose, arabinose, xylose, mannose, glucose, and galactose, were analyzed. It is well known that the main components of NRS are cellulose, hemicelluloses and lignin. Data of neutral sugar contents for NRS promoted that the principal component of NRS is cellulose (43.64%), followed by hemicellulose (20.84%). The rest species were lignin and other ingredients. After alkali treatment, rhamnose disappeared, and contents of other neutral sugars decreased except for glucose. The content of glucose in ARS was 93.28%, and the content of xylose decreased from 20.84% to 3.49%. These changes suggested that the dominant component of ARS was cellulose, most hemicellulose, lignin and other ingredients had been removed by alkali treatment. All contents of remained monosaccharides decreased with etherification reaction time. The content of glucose reduced to 31.14%, suggesting that the chemical structure of cellulose in rice straw was destroyed by etherification. After modification for 2 h, mannose and galactose were completely removed. After modification for 5 h, the contents of residual monosaccharides, i.e., glucose, xylose and arabinose, changed very little. These phenomena supported the results of FT-IR that the etherification process was finished within 5 h.

**Table 2:** Contents of neutral sugars in hydrolysis products of NRS, ARS and ERS.

Dry Samples	Contents of neutral sugars of hydrolysis products (%(w/w))						
	glucose	xylose	arabinose	mannose	galactose	rhamnose	total sugars
NRS	43.64	20.84	3.08	0.62	1.67	0.16	70.01
ARS	93.28	3.49	0.59	0.26	0.43	0.00	98.05
ERS (1 h)	66.35	1.63	0.28	0.17	0.47	0.00	68.90
ERS (2 h)	37.34	1.59	0.11	0.00	0.00	0.00	39.03
ERS (3 h)	37.19	0.96	0.04	0.00	0.00	0.00	38.19
ERS (4 h)	34.21	1.16	0.09	0.00	0.00	0.00	35.46
ERS (5 h)	33.31	1.13	0.08	0.00	0.00	0.00	34.52
ERS (6 h)	32.52	1.11	0.07	0.00	0.00	0.00	33.70
ERS (7 h)	31.49	1.16	0.05	0.00	0.00	0.00	32.71
ERS (8 h)	31.14	1.15	0.15	0.00	0.00	0.00	32.44

### Mechanism of etherification with EPI

**Figure 3:**
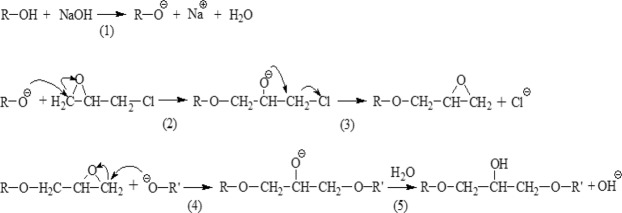
Reaction mechanism of etherification of rice straw cellulose by epichlorohydrin.

Based on FT-IR spectra, ^13^C-NMR, elemental analysis and neutral sugar analysis, mechanism of etherification with EPI for rice straw was speculated as shown in Fig. 3. The etherification process was divided into three stages. In the first stage, cellulose was alkalized by NaOH to produce sodium cellulosate (reaction 1). The second stage contained reaction (2) and (3), the third stage contained reaction (4) and (5). The epoxy ring was attacked by oxygen atom of straw cellulose at C-2 (or C-3, C-5 and C-6), leading the epoxy ring to open. Thus, an epoxy unit was introduced into structure of the rice straw (reaction 2). At the same time, the C–Cl bond broke and a new epoxy ring formed (reaction 3). In the third stage, another oxygen atom of cellulosate attacked the end C of the newly formed (in the second stage) epoxy ring and made it open and a negatively charged oxygen atom was produced (reaction 4). In the last step, the negative oxygen atom formed a new hydroxyl group by capturing a H^+^ from water (reaction 5). During this etherification process, two hydroxyl groups were replaced by two ether bonds, while a new hydroxyl group was formed in the last step. As a result, the number of hydroxyl group in rice straw was reduced, and it could be expected that the hydrophilicity of ERS decreased.

## Conclusion

After alkali treatment, most of hemicellulose, lignin and other ingredients in NRS were removed, and the dominant component of ARS was cellulose. By etherification with EPI, C_3_H_6_O unit was introduced into the structure of cellulose, and the contents of C and H of ERS increased. The etherification reaction occurred at the hydroxyl sites of cellulose’s C-2, C-3, C-5 and C-6, and finished within 5 h. The etherification process in alkaline medium consisted of three stages. The number of hydroxyl group in ERS was reduced, and the hydrophilicity of ERS might be decreased.
